# Comprehensive clinical application analysis of artificial intelligence-enabled electrocardiograms for screening multiple valvular heart diseases

**DOI:** 10.18632/aging.205835

**Published:** 2024-05-16

**Authors:** Yu-Ting Lin, Chin-Sheng Lin, Chien-Sung Tsai, Dung-Jang Tsai, Yu-Sheng Lou, Wen-Hui Fang, Yung-Tsai Lee, Chin Lin

**Affiliations:** 1 Department of Surgery, Division of Cardiovascular Surgery, Tri-Service General Hospital, National Defense Medical Center, Taipei, Taiwan; 2 Department of Internal Medicine, Division of Cardiology, Tri-Service General Hospital, National Defense Medical Center, Taipei, Taiwan; 3 Artificial Intelligence of Things Center, Tri-Service General Hospital, National Defense Medical Center, Taipei, Taiwan; 4 Graduate Institutes of Life Sciences, National Defense Medical Center, Taipei, Taiwan; 5 Department of Family and Community Medicine, Tri-Service General Hospital, National Defense Medical Center, Taipei, Taiwan; 6 Division of Cardiovascular Surgery, Cheng Hsin Rehabilitation and Medical Center, Taipei, Taiwan; 7 Medical Technology Education Center, School of Medicine, National Defense Medical Center, Taipei, Taiwan; 8 Graduate Institute of Aerospace and Undersea Medicine, National Defense Medical Center, Taipei, Taiwan; 9 Department of Statistics and Information Science, Fu Jen Catholic University, New Taipei City, Taiwan; 10 Department of Exercise and Healthy Science, National Taipei University of Nursing and Healthy Science, Taipei, Taiwan

**Keywords:** artificial intelligence, electrocardiogram, transthoracic echocardiography, deep learning, valvular heart disease

## Abstract

Background: Valvular heart disease (VHD) is becoming increasingly important to manage the risk of future complications. Electrocardiographic (ECG) changes may be related to multiple VHDs, and (AI)-enabled ECG has been able to detect some VHDs. We aimed to develop five deep learning models (DLMs) to identify aortic stenosis, aortic regurgitation, pulmonary regurgitation, tricuspid regurgitation, and mitral regurgitation.

Methods: Between 2010 and 2021, 77,047 patients with echocardiography and 12-lead ECG performed within 7 days were identified from an academic medical center to provide DLM development (122,728 ECGs), and internal validation (7,637 ECGs). Additional 11,800 patients from a community hospital were identified to external validation. The ECGs were classified as with or without moderate-to-severe VHDs according to transthoracic echocardiography (TTE) records, and we also collected the other echocardiographic data and follow-up TTE records to identify new-onset valvular heart diseases.

Results: AI-ECG adjusted for age and sex achieved areas under the curves (AUCs) of >0.84, >0.80, >0.77, >0.83, and >0.81 for detecting aortic stenosis, aortic regurgitation, pulmonary regurgitation, tricuspid regurgitation, and mitral regurgitation, respectively. Since predictions of each DLM shared similar components of ECG rhythms, the positive findings of each DLM were highly correlated with other valvular heart diseases. Of note, a total of 37.5–51.7% of false-positive predictions had at least one significant echocardiographic finding, which may lead to a significantly higher risk of future moderate-to-severe VHDs in patients with initially minimal-to-mild VHDs.

Conclusion: AI-ECG may be used as a large-scale screening tool for detecting VHDs and a basis to undergo an echocardiography.

## INTRODUCTION

The cardiac blood flow direction from one area to another is related to heart valves, including the aortic valve, pulmonary valve, tricuspid valve, and mitral valve. In the United States, 2.5% of patients suffered from moderate or severe valvular heart disease, [1] and more than half were asymptomatic. Moreover, aortic stenosis was also a significant valvular heart disease and was present in 0.4% of patients, [1] and valvular heart diseases were more common in elderly individuals. Severe valvular heart disease may lead to heart failure and sudden death, [2] and immediate intervention is necessary to manage the risk of complications [3]. Currently, most asymptomatic patients with valvular heart disease are identified by advanced health examination, including echocardiography. Due to the characteristics of echocardiography of expensiveness and requirement of indispensable specialists, it cannot be used as a wider screening tool, and a universally available alternative to screen for potential valvular diseases is needed.

Since valvular heart diseases are related to ventricular hypertrophy, atrium enlargement, atrial fibrillation, atrial premature complex and ventricular premature complex, changes in electrocardiography (ECG) were observed in patients with those conditions. With the revolution of deep learning models (DLMs), artificial intelligence (AI)-enabled ECG may extract subtle rhythm abnormalities beyond those extracted by human experts to identify diverse cardiac diseases [4]. Previous studies have already developed a DLM to identify left ventricular hypertrophy, [5] left atrium enlargement, [6] and arrhythmia [7] using available large annotation databases. We hypothesized that AI-ECG would allow for the detection of valvular diseases in individuals with at least cardiac structure or rhythm changes.

Previous studies have developed DLMs for detecting aortic stenosis with an AUC >0.86 using 12-lead ECG and demography; [8, 9] aortic regurgitation with an AUC >0.80 using 12-lead ECG and demography; [10] and mitral regurgitation with an AUC >0.81 using 12-lead ECG [11]. However, the low positive predictive value, which may cause anxiety and inconvenience for patients, was the major concern for direct application of these DLMs in clinical practice. Previous research on false-positive prediction by AI-ECG of left ventricular dysfunction (LVD) with more than 4-fold risk of new-onset LVD, [4] also showed that false-positive cases might be considered at high risk of new-onset aortic stenosis [8] and mitral regurgitation [11]. However, the mechanism of this phenomenon is still unclear, leading to a lack of strategies for intervention. Moreover, a growing number of DLMs for detecting more valvular diseases in one AI-ECG report may lead to confusion. A comprehensive clinical application analysis to simultaneously consider multiple valvular heart diseases should be conducted before using Al-ECG in real-world clinical practice.

This study has three objectives: (i) to extensively explore the ability of AI-ECG to detect more valvular heart diseases; (ii) to develop a strategy to interpret the AI-ECG results with multiple predictions for further intervention recommendations; and (iii) to assess the prognostic performance of AI-ECG in individuals without significant valvular heart diseases.

## RESULTS

Table 1 shows the patient characteristics in the development set, tuning set, internal validation set and external validation set. The prevalence rates of moderate-to-severe aortic stenosis, aortic regurgitation, pulmonary regurgitation, tricuspid regurgitation, and mitral regurgitation in the internal/external validation sets were 0.6%/0.9%, 4.8%/5.5%, 1.9%/2.1%, 12.6%/13.6%, and 10.8%/11.1%, respectively. In summary, the patients in the external validation set were older and had more comorbidities than those in the internal validation set.

**Table 1:** Baseline characteristics.

	**Development set**	**Tuning set**	**Internal validation set**	**External validation set**
**Valvular diseases**				
Aortic stenosis				
*minimal*	99,097 (97.1%)	19,677 (95.3%)	7,461 (97.7%)	11,419 (96.8%)
*mild*	1,877 (1.8%)	637 (3.1%)	126 (1.7%)	270 (2.3%)
*moderate*	684 (0.7%)	224 (1.1%)	30 (0.4%)	73 (0.6%)
*severe*	427 (0.4%)	105 (0.5%)	19 (0.2%)	38 (0.3%)
Aortic regurgitation				
*minimal*	69,538 (68.1%)	12,250 (59.3%)	5,310 (69.5%)	7,921 (67.1%)
*mild*	26,859 (26.3%)	6,811 (33.0%)	1,954 (25.6%)	3,236 (27.4%)
*moderate*	5,146 (5.0%)	1,382 (6.7%)	331 (4.3%)	600 (5.1%)
*severe*	542 (0.5%)	200 (1.0%)	41 (0.5%)	43 (0.4%)
Pulmonary regurgitation				
*minimal*	77,466 (75.9%)	14,702 (71.2%)	5,857 (76.7%)	8,957 (75.9%)
*mild*	22,346 (21.9%)	5,267 (25.5%)	1,638 (21.5%)	2,594 (22.0%)
*moderate*	2,105 (2.1%)	643 (3.1%)	137 (1.8%)	230 (1.9%)
*severe*	168 (0.2%)	31 (0.2%)	4 (0.1%)	19 (0.2%)
Tricuspid regurgitation				
*minimal*	3,8902 (38.1%)	6,556 (31.8%)	3,194 (41.8%)	4,768 (40.4%)
*mild*	4,7831 (46.9%)	9,933 (48.1%)	3,481 (45.6%)	5,417 (45.9%)
*moderate*	1,1962 (11.7%)	3,075 (14.9%)	781 (10.2%)	1,267 (10.7%)
*severe*	3,390 (3.3%)	1,079 (5.2%)	180 (2.4%)	348 (2.9%)
Mitral regurgitation				
*minimal*	48,860 (47.9%)	8,419 (40.8%)	3,921 (51.3%)	5,867 (49.7%)
*mild*	39,748 (38.9%)	8,570 (41.5%)	2,890 (37.8%)	4,625 (39.2%)
*moderate*	10,817 (10.6%)	2,899 (14.0%)	680 (8.9%)	1,104 (9.4%)
*severe*	2,660 (2.6%)	755 (3.7%)	145 (1.9%)	204 (1.7%)
**Demography**				
Sex (male)	52,421 (53.8%)	10,913 (52.9%)	3,871 (50.7%)	5,861 (49.7%)
Age (years)	64.0 ± 17.4	68.1 ± 16.3	63.4 ± 16.6	65.8 ± 18.1
BMI (kg/m^2^)	24.6 ± 4.4	24.3 ± 4.4	24.5 ± 4.4	24.4 ± 4.3
**Disease history**				
DM	23,111 (23.7%)	7,394 (35.8%)	2,259 (29.6%)	3,654 (31.0%)
HTN	39,229 (40.3%)	12,018 (58.2%)	3,964 (51.9%)	6,506 (55.1%)
HLP	29,152 (29.9%)	9,256 (44.8%)	3,129 (41.0%)	5,198 (44.1%)
CKD	23,473 (24.1%)	9,036 (43.8%)	1,835 (24.0%)	2,892 (24.5%)
CAD	27,043 (27.8%)	8,429 (40.8%)	2,351 (30.8%)	3,649 (30.9%)
HF	12,830 (13.2%)	4,865 (23.6%)	934 (12.2%)	1,476 (12.5%)
Afib	6,550 (6.7%)	2,628 (12.7%)	491 (6.4%)	752 (6.4%)
COPD	12,296 (12.6%)	4,513 (21.9%)	1,503 (19.7%)	2,777 (23.5%)
**Echocardiography data**				
EF (%)	63.4 ± 12.7	61.0 ± 14.3	65.2 ± 11.4	65.4 ± 10.8
LV-D (mm)	47.5 ± 7.1	47.9 ± 7.8	47.3 ± 7.1	47.1 ± 6.8
LV-S (mm)	30.3 ± 6.9	31.2 ± 7.8	29.8 ± 6.8	29.6 ± 6.3
IVS (mm)	11.2 ± 2.7	11.6 ± 2.7	11.2 ± 2.8	11.1 ± 2.8
LVPW (mm)	9.3 ± 1.7	9.5 ± 1.8	9.3 ± 1.7	9.1 ± 1.7
LA (mm)	38.4 ± 7.5	39.6 ± 8.1	38.5 ± 7.6	38.7 ± 7.3
AO (mm)	32.7 ± 4.4	33.1 ± 4.4	32.9 ± 4.5	32.8 ± 4.3
RV (mm)	23.8 ± 5.0	24.2 ± 5.1	24.2 ± 5.0	24.0 ± 4.9
PASP (mmHg)	33.2 ± 11.1	34.6 ± 12.4	32.1 ± 10.4	33.0 ± 10.7
PE (mm)	0.5 ± 2.1	0.6 ± 2.1	0.3 ± 1.8	0.4 ± 1.7

Abbreviations: BMI: body mass index; DM: diabetes mellitus; HTN: hypertension; HLP: hyperlipidemia; CKD: chronic kidney disease; CAD: coronary artery disease; HF: heart failure; Afib: atrial fibrillation; COPD: chronic obstructive pulmonary disease; EF: ejection fraction; LV-D: left ventricle (end-diastole); LV-S: left ventricle (end-systole); IVS: inter-ventricular septum; LVPW: left ventricular posterior wall; LA: left atrium; AO: aortic root; RV: right ventricle; PASP: pulmonary artery systolic pressure; PE: pericardial effusion.

**Figure 1:**
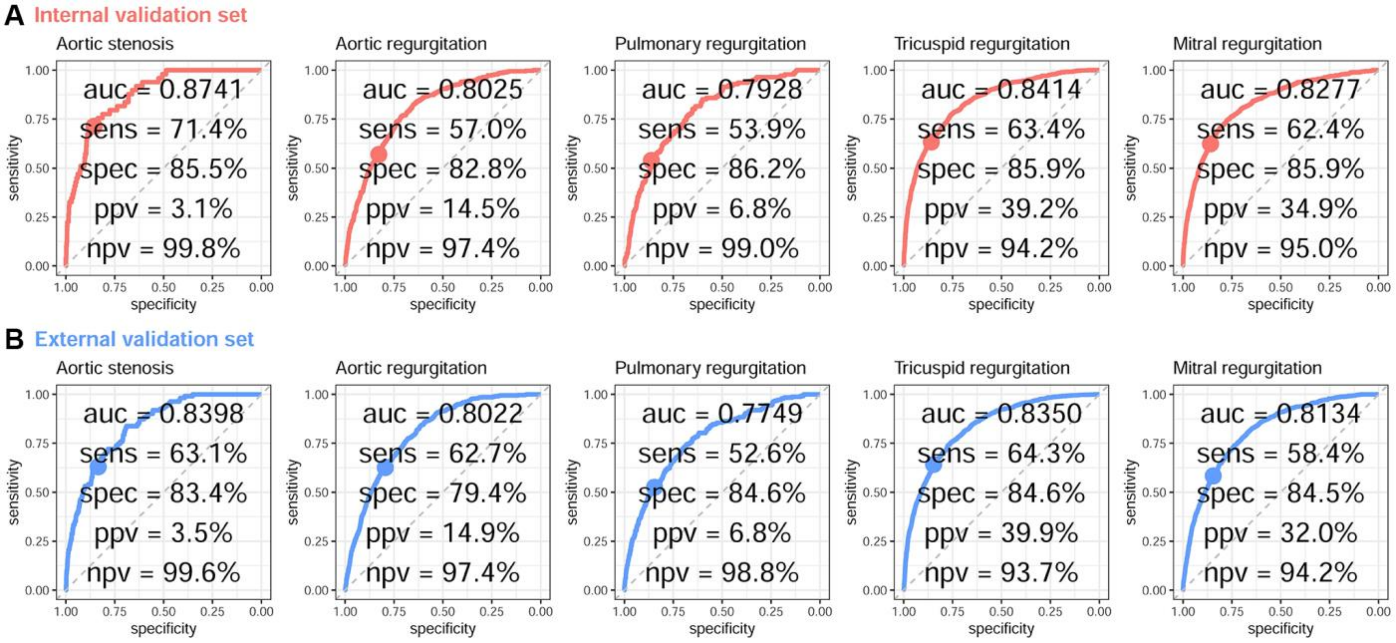
**ROC curve analysis for VHD from a DLM based on age, sex, and ECG voltage–time traces.** The receiver operating characteristic (ROC) curve (x-axis = specificity and y-axis = sensitivity) and area under the ROC curve (AUC) were calculated using the internal validation set (**A**) and external validation set (**B**). The operating point was selected based on the maximum Youden’s index in the tuning set, which was used for calculating the corresponding sensitivities and specificities in the two validation sets.

The algorithms performed well in identifying each valvular disease in the validation datasets (Table 2). The DLM using ECG alone achieved AUCs of 0.768–0.847 in internal validation and 0.763–0.827 in external validation. The AUCs were 0.002 to 0.034 higher using age and sex. Using the operating point with equal sensitivity and specificity for the integration of age, sex, and ECG, Figure 1 shows sensitivities of 63.1–71.4% and specificities of 84.3–85.5% for detecting moderate-to-severe aortic stenosis; sensitivities of 58.1–63.5% and specificities of 79.1–82.4% for detecting aortic regurgitation; sensitivities of 54.6–58.2% and specificities of 82.0–82.9% for detecting pulmonary regurgitation; sensitivities of 62.2–63.3% and specificities of 85.3–86.6% for detecting tricuspid regurgitation; and sensitivities of 59.5–63.5% and specificities of 84.1–85.5% for detecting mitral regurgitation. Compared with the near perfect negative predictive values of more than 94.3% in each analysis, the related low positive predictive values ranging from 3.1% to 40.6% may be the major concern in AI-ECG for screening valvular diseases.

**Table 2:** Detailed model performance for detecting each valvular disease.

	**Sensitivity**	**Specificity**	**PPV**	**NPV**	**AUC**
**Aortic stenosis**					
ECG alone (internal)	0.714 (0.588–0.841)	0.798 (0.705–0.807)	0.022 (0.015–0.030)	0.998 (0.996–0.999)	0.847 (0.796–0.898)
ECG alone (external)	0.613 (0.522–0.703)	0.796 (0.605–0.803)	0.028 (0.021–0.034)	0.995 (0.994–0.997)	0.812 (0.775–0.848)
Age, sex + ECG (internal)	0.714 (0.588–0.841)	0.855 (0.706–0.863)	0.031 (0.021–0.041)	0.998 (0.997–0.999)	0.874 (0.834–0.915)
Age, sex + ECG (external)	0.631 (0.541–0.720)	0.834 (0.624–0.841)	0.035 (0.027–0.043)	0.996 (0.995–0.997)	0.840 (0.808–0.872)
**Aortic regurgitation**					
ECG alone (internal)	0.616 (0.566–0.665)	0.754 (0.606–0.764)	0.114 (0.100–0.128)	0.975 (0.970–0.979)	0.768 (0.745–0.791)
ECG alone (external)	0.642 (0.605–0.679)	0.752 (0.634–0.760)	0.130 (0.118–0.141)	0.973 (0.970–0.977)	0.773 (0.756–0.790)
Age, sex + ECG (internal)	0.570 (0.520–0.620)	0.828 (0.561–0.836)	0.145 (0.127–0.163)	0.974 (0.970–0.978)	0.802 (0.781–0.824)
Age, sex + ECG (external)	0.627 (0.589–0.664)	0.794 (0.619–0.802)	0.149 (0.136–0.163)	0.974 (0.970–0.977)	0.802 (0.787–0.817)
**Pulmonary regurgitation**					
ECG alone (internal)	0.511 (0.428–0.593)	0.866 (0.503–0.873)	0.067 (0.052–0.082)	0.989 (0.987–0.992)	0.774 (0.736–0.812)
ECG alone (external)	0.478 (0.416–0.540)	0.863 (0.472–0.869)	0.070 (0.058–0.082)	0.987 (0.985–0.989)	0.763 (0.733–0.793)
Age, sex + ECG (internal)	0.539 (0.457–0.621)	0.862 (0.531–0.870)	0.068 (0.054–0.083)	0.990 (0.988–0.992)	0.793 (0.758–0.828)
Age, sex + ECG (external)	0.526 (0.464–0.588)	0.846 (0.520–0.852)	0.068 (0.057–0.080)	0.988 (0.986–0.990)	0.775 (0.746–0.804)
**Tricuspid regurgitation**					
ECG alone (internal)	0.656 (0.626–0.686)	0.838 (0.647–0.847)	0.368 (0.345–0.391)	0.944 (0.938–0.950)	0.833 (0.819–0.847)
ECG alone (external)	0.652 (0.629–0.675)	0.826 (0.645–0.833)	0.373 (0.355–0.391)	0.937 (0.932–0.942)	0.827 (0.817–0.838)
Age, sex + ECG (internal)	0.634 (0.603–0.664)	0.859 (0.625–0.867)	0.392 (0.368–0.416)	0.942 (0.936–0.948)	0.841 (0.828–0.855)
Age, sex + ECG (external)	0.643 (0.619–0.666)	0.846 (0.636–0.853)	0.399 (0.380–0.418)	0.937 (0.932–0.942)	0.835 (0.825–0.845)
**Mitral regurgitation**					
ECG alone (internal)	0.611 (0.578–0.644)	0.864 (0.603–0.872)	0.352 (0.327–0.377)	0.948 (0.943–0.954)	0.824 (0.809–0.840)
ECG alone (external)	0.563 (0.537–0.590)	0.855 (0.557–0.861)	0.326 (0.307–0.345)	0.940 (0.935–0.945)	0.811 (0.799–0.823)
Age, sex + ECG (internal)	0.624 (0.591–0.657)	0.859 (0.616–0.867)	0.349 (0.325–0.373)	0.950 (0.944–0.955)	0.828 (0.813–0.843)
Age, sex + ECG (external)	0.584 (0.557–0.611)	0.845 (0.577–0.852)	0.320 (0.301–0.338)	0.942 (0.937–0.947)	0.813 (0.802–0.825)

Abbreviations: PPV: positive predictive value; NPV: negative predictive value; AUC: area under receiver operating characteristic curve.

Table 3 presents the analysis results for different levels of disease severity. We found that AI-ECG exhibits higher sensitivities in detecting 5 severe valvular diseases compared to moderate valvular diseases, ranging from 5.2% to 31.4%. After incorporating sex and age information, this increased range of sensitivities from −5.6% to 47.4%. All AUCs for detecting severe cases are higher compared to detecting moderate cases, indicating that AI-ECG is less likely to miss severe cases. The results of the stratified analysis based on demography and disease history are presented in Table 4. We found that AI-ECG exhibits a reduction of over 10% in AUC for detecting aortic stenosis in individuals with a history of chronic kidney disease (CKD) and atrial fibrillation (Afib). Additionally, there is a 10% decrease in AUC for aortic regurgitation and mitral regurgitation in patients over 65 years old. This stratified analysis reveals limitations in the application of AI-ECG for elderly with those histories.

**Table 3:** Stratified analysis of disease severity for detecting each valvular disease.

	**ECG alone Specificity AUC**			**Age, sex + ECG**		
	**Sensitivity**	**Specificity**	**AUC**	**Sensitivity**	**Specificity**	**AUC**
**Aortic stenosis**						
Moderate (internal)	0.667 (0.498–0.835)	0.798 (0.658–0.807)	0.811 (0.734–0.888)	0.667 (0.498–0.835)	0.855 (0.659–0.863)	0.852 (0.791–0.912)
Moderate (external)	0.562 (0.448–0.675)	0.796 (0.554–0.803)	0.783 (0.736–0.830)	0.562 (0.448–0.675)	0.834 (0.555–0.841)	0.816 (0.778–0.855)
Severe (internal)	0.789 (0.606–0.973)	0.798 (0.780–0.807)	0.904 (0.863–0.945)	0.789 (0.606–0.973)	0.855 (0.782–0.863)	0.909 (0.870–0.948)
Severe (external)	0.711 (0.566–0.855)	0.796 (0.703–0.803)	0.867 (0.813–0.920)	0.763 (0.628–0.898)	0.834 (0.756–0.841)	0.885 (0.833–0.937)
**Aortic regurgitation**						
Moderate (internal)	0.604 (0.552–0.657)	0.754 (0.594–0.764)	0.759 (0.735–0.783)	0.547 (0.493–0.600)	0.828 (0.538–0.836)	0.799 (0.777–0.821)
Moderate (external)	0.625 (0.586–0.664)	0.752 (0.617–0.760)	0.765 (0.747–0.782)	0.613 (0.574–0.652)	0.794 (0.606–0.802)	0.797 (0.781–0.813)
Severe (internal)	0.707 (0.568–0.847)	0.754 (0.697–0.764)	0.843 (0.783–0.903)	0.756 (0.625–0.888)	0.828 (0.747–0.836)	0.831 (0.763–0.900)
Severe (external)	0.884 (0.788–0.980)	0.752 (0.876–0.760)	0.885 (0.837–0.932)	0.814 (0.698–0.930)	0.794 (0.806–0.802)	0.874 (0.821–0.927)
**Pulmonary regurgitation**						
Moderate (internal)	0.504 (0.420–0.587)	0.866 (0.496–0.873)	0.771 (0.732–0.810)	0.526 (0.442–0.609)	0.862 (0.518–0.870)	0.788 (0.753–0.824)
Moderate (external)	0.474 (0.409–0.538)	0.863 (0.468–0.869)	0.760 (0.729–0.792)	0.530 (0.466–0.595)	0.846 (0.524–0.852)	0.772 (0.741–0.802)
Severe (internal)	0.750 (0.326–1.174)	0.866 (0.742–0.873)	0.896 (0.822–0.971)	1.000 (1.000–1.000)	0.862 (0.992–0.870)	0.940 (0.886–0.994)
Severe (external)	0.526 (0.302–0.751)	0.863 (0.520–0.869)	0.802 (0.713–0.892)	0.474 (0.249–0.698)	0.846 (0.467–0.852)	0.814 (0.729–0.900)
**Tricuspid regurgitation**						
Moderate (internal)	0.597 (0.562–0.631)	0.838 (0.588–0.847)	0.807 (0.791–0.823)	0.576 (0.542–0.611)	0.859 (0.568–0.867)	0.817 (0.801–0.832)
Moderate (external)	0.589 (0.562–0.616)	0.826 (0.581–0.833)	0.800 (0.787–0.812)	0.576 (0.549–0.603)	0.846 (0.569–0.853)	0.808 (0.796–0.820)
Severe (internal)	0.911 (0.870–0.953)	0.838 (0.902–0.847)	0.946 (0.932–0.960)	0.883 (0.836–0.930)	0.859 (0.875–0.867)	0.949 (0.936–0.962)
Severe (external)	0.882 (0.848–0.916)	0.826 (0.875–0.833)	0.929 (0.916–0.942)	0.885 (0.852–0.919)	0.846 (0.878–0.853)	0.933 (0.921–0.945)
**Mitral regurgitation**						
Moderate (internal)	0.565 (0.527–0.602)	0.864 (0.557–0.872)	0.804 (0.786–0.821)	0.579 (0.542–0.617)	0.859 (0.571–0.867)	0.808 (0.791–0.825)
Moderate (external)	0.523 (0.493–0.552)	0.855 (0.516–0.861)	0.795 (0.782–0.808)	0.541 (0.511–0.570)	0.845 (0.534–0.852)	0.798 (0.785–0.810)
Severe (internal)	0.828 (0.766–0.889)	0.864 (0.819–0.872)	0.920 (0.901–0.940)	0.834 (0.774–0.895)	0.859 (0.826–0.867)	0.920 (0.899–0.940)
Severe (external)	0.784 (0.728–0.841)	0.855 (0.778–0.861)	0.899 (0.878–0.921)	0.819 (0.766–0.872)	0.845 (0.812–0.852)	0.899 (0.877–0.920)

All analyses shared the same control group (minimal to mild) for each valvular disease. It is important to emphasize that due to the exclusion of certain severity cases, the positive predictive value and negative predictive value hold no significance in this analysis. Abbreviations: AUC: area under receiver operating characteristic curve.

**Table 4:** Stratified analysis of demography and disease history for detecting each valvular disease using ECG alone.

	**Aortic stenosis**	**Aortic regurgitation**	**Pulmonary regurgitation**	**Tricuspid regurgitation**	**Mitral regurgitation**
**Demography**					
Female	0.820 (0.772–0.869)	0.763 (0.736–0.790)	0.760 (0.717–0.804)	0.805 (0.789–0.820)	0.802 (0.785–0.818)
Male	0.802 (0.745–0.859)	0.778 (0.756–0.800)	0.768 (0.728–0.808)	0.853 (0.839–0.867)	0.825 (0.807–0.843)
Age <65 years	0.892 (0.844–0.939)	0.810 (0.764–0.855)	0.707 (0.623–0.790)	0.850 (0.829–0.872)	0.853 (0.831–0.875)
Age ≥65 years	0.751 (0.700–0.802)	0.707 (0.686–0.729)	0.723 (0.688–0.759)	0.778 (0.763–0.792)	0.757 (0.741–0.773)
BMI <24.0 kg/m^2^	0.795 (0.742–0.849)	0.783 (0.761–0.805)	0.759 (0.720–0.799)	0.812 (0.798–0.827)	0.810 (0.793–0.826)
BMI ≥24.0 kg/m^2^	0.829 (0.778–0.879)	0.758 (0.732–0.784)	0.764 (0.718–0.810)	0.843 (0.828–0.858)	0.812 (0.795–0.829)
**Disease history**					
Without DM	0.815 (0.774–0.856)	0.800 (0.781–0.818)	0.764 (0.729–0.800)	0.835 (0.823–0.848)	0.822 (0.808–0.836)
With DM	0.814 (0.740–0.888)	0.703 (0.665–0.742)	0.771 (0.717–0.824)	0.813 (0.794–0.833)	0.788 (0.767–0.810)
Without HTN	0.814 (0.739–0.888)	0.795 (0.766–0.823)	0.752 (0.698–0.806)	0.835 (0.818–0.852)	0.823 (0.803–0.842)
With HTN	0.789 (0.745–0.834)	0.747 (0.725–0.770)	0.758 (0.721–0.794)	0.817 (0.803–0.831)	0.795 (0.779–0.810)
Without HLP	0.824 (0.778–0.869)	0.789 (0.768–0.809)	0.772 (0.736–0.809)	0.835 (0.822–0.848)	0.824 (0.809–0.839)
With HLP	0.796 (0.735–0.857)	0.744 (0.715–0.774)	0.748 (0.696–0.800)	0.816 (0.798–0.833)	0.794 (0.774–0.813)
Without CKD	0.837 (0.791–0.883)	0.781 (0.759–0.802)	0.759 (0.720–0.798)	0.832 (0.819–0.845)	0.822 (0.807–0.837)
With CKD	0.718 (0.648–0.788)	0.720 (0.689–0.751)	0.730 (0.678–0.781)	0.783 (0.763–0.803)	0.747 (0.725–0.770)
Without CAD	0.836 (0.793–0.879)	0.776 (0.756–0.797)	0.788 (0.754–0.822)	0.827 (0.814–0.840)	0.822 (0.808–0.836)
With CAD	0.764 (0.697–0.830)	0.765 (0.734–0.795)	0.705 (0.646–0.764)	0.829 (0.810–0.848)	0.787 (0.765–0.809)
Without HF	0.813 (0.770–0.856)	0.765 (0.745–0.785)	0.744 (0.709–0.780)	0.813 (0.800–0.825)	0.791 (0.777–0.806)
With HF	0.737 (0.648–0.827)	0.728 (0.692–0.765)	0.760 (0.702–0.818)	0.817 (0.793–0.840)	0.786 (0.761–0.811)
Without Afib	0.815 (0.774–0.855)	0.770 (0.751–0.788)	0.742 (0.708–0.776)	0.810 (0.798–0.822)	0.804 (0.791–0.817)
With Afib	0.665 (0.533–0.797)	0.690 (0.637–0.743)	0.747 (0.677–0.818)	0.762 (0.728–0.796)	0.707 (0.667–0.747)
Without COPD	0.834 (0.795–0.873)	0.786 (0.767–0.806)	0.763 (0.728–0.799)	0.835 (0.822–0.847)	0.827 (0.814–0.841)
With COPD	0.753 (0.669–0.837)	0.728 (0.694–0.761)	0.760 (0.706–0.814)	0.800 (0.778–0.821)	0.759 (0.732–0.785)

All results were presented using area under receiver operating characteristic curve (AUC). Abbreviations: BMI: body mass index; DM: diabetes mellitus; HTN: hypertension; HLP: hyperlipidemia; CKD: chronic kidney disease; CAD: coronary artery disease; HF: heart failure; Afib: atrial fibrillation; COPD: chronic obstructive pulmonary disease.

**Figure 2:**
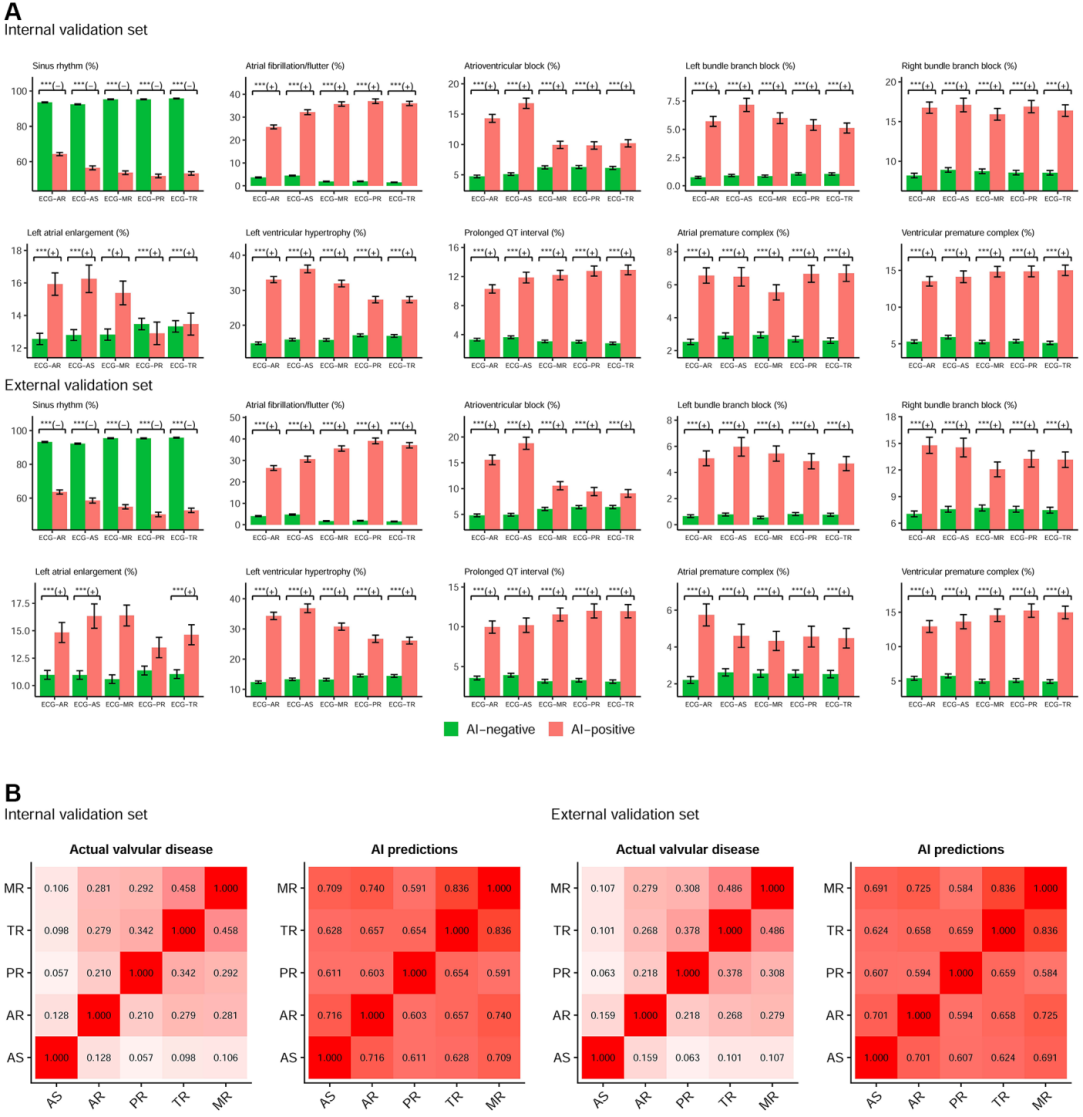
**The components of AI predictions for detecting each valvular disease.** (**A**) Relationship between ECG-screened valvular diseases and ECG rhythms. The plots display two groups, positive (AI-positive) and negative (AI-negative) findings, by the ECG networks using ECG alone. Sinus rhythm is associated with AI-negative (green bar), and other abnormal rhythms are associated with AI-positive (red bar). Abbreviations: ^*^p < 0.05; ^**^p < 0.01; ^***^p < 0.001. The +/− demonstrates the positive/negative relationship. (**B**) The relationship between each valvular disease in actual status and prediction. The values in each cell are the Spearman correlation coefficients.

Figure 2A shows the relationship between ECG-screened valvular diseases and ECG rhythms. Positive ECGs had a lower prevalence of sinus rhythm and a higher prevalence of atrial fibrillation/flutter, atrioventricular block, left bundle branch block, right bundle branch block, left atrial enlargement, left ventricular hypertrophy, prolonged QT interval, atrial premature complex, and ventricular premature complex than those classified as negative by each DLM. High consistency in the rhythm difference in the DLM for detecting each valvular disease revealed similar components to identify positive ECGs, which implies that these positive predictions may be related to other valvular diseases. Figure 2B validated the hypothesis that predictions between DLMs for detecting each valvular disease were highly correlated (ranging from 0.584 to 0.836), although the correlation was low in actual valvular diseases (ranging from 0.057 to 0.486). Moreover, more abnormal ECG rhythms in positive cases may also be related to other cardiac comorbidities and complications.

**Figure 3:**
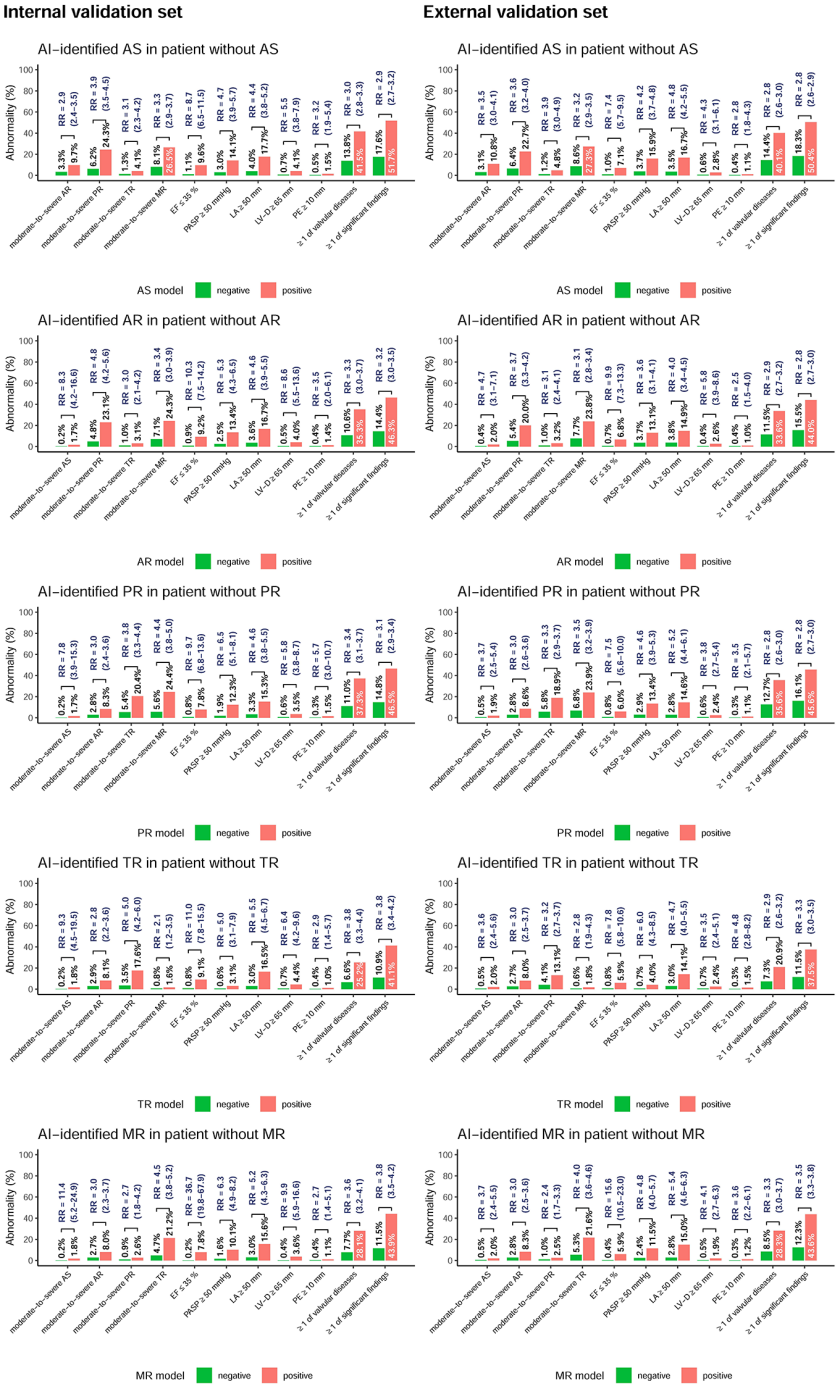
**Prevalence (*p*) of echocardiographic abnormalities in patients stratified by each AI classification using ECG alone.** The plots display the abnormal prevalence in the two groups, including positive and negative findings based on ECG. The ≥1 of valvular diseases was defined as at least 1 moderate-to-severe valvular disease, and the ≥1 of significant findings was defined as at least 1 abnormal echocardiographic finding. The relative risk (RR) was calculated as (*p*_AI-positive/_*p*_AI-negative_) and is presented with the associated 95% confidence interval.

We further analyzed echocardiographic abnormalities in false-positive cases compared to the true negative cases in Figure 3. For the patients without aortic stenosis in the internal validation set, the false-positive cases had a 2.9-to-3.9-fold risk of presenting other valvular diseases, and there were 41.5% false-positive cases with at least 1 other valvular disease. Moreover, these false-positive cases also presented worse cardiac function and more anomalies: 8.7-fold risk of low ejection fraction, 4.7-fold risk of high pulmonary artery systolic pressure, 4.4-fold risk of left atrial enlargement, 5.5-fold risk of larger left ventricular end-diastolic diameter, and 3.2-fold risk of significant pericardial effusion. In summary, more than 50% of false-positive cases presented at least 1 significant echocardiographic abnormality, and this phenomenon was also validated in external validation. Similar trends were shown in all false-positive ECGs by DLM for detecting aortic regurgitation, pulmonary regurgitation, tricuspid regurgitation, and mitral regurgitation. False-positive cases had a higher risk of every kind of echocardiographic abnormality, and more than 37.5% of them had more than 1 significant echocardiographic abnormality, which revealed the importance of conducting echocardiography for AI-identified positive cases.

**Figure 4:**
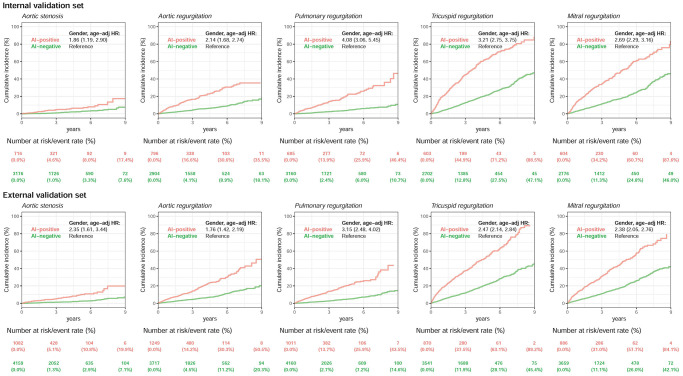
**Long-term incidence of developing severity stratified by AI classification using ECG alone.** Long-term incidence of developing each moderate-to-severe valvular disease in patients with initially minimal-to-mild valvular diseases stratified by AI classification using ECG alone. Long-term outcome of patients with echocardiographic minimal-to-mild valvular diseases at the time of initial classification, stratified by the initial network classification. The ordinate shows the cumulative incidence of developing moderate-to-severe valvular diseases, and the abscissa indicates years from the time of index ECG–TTE evaluation. A significantly higher risk of future moderate-to-severe valvular diseases was present when the AI algorithm defined the ECG as positive compared with patients with minimal-to-mild valvular diseases who were classified as having a negative finding by the ECG network. The analyses were conducted in both internal and external validation sets. The table shows the at-risk population and cumulative risk for the given time intervals in each risk stratification.

We followed more than 3,300/4,300 initially echo-negative patients with ≥2 ECG–TTE pairs in the internal/external validation sets (Figure 4). For patients in the internal validation sets without corresponding valvular diseases initially and more than 9 years of follow-up, the cumulative incidence rates in positive/negative cases for new-onset aortic stenosis, aortic regurgitation, pulmonary regurgitation, tricuspid regurgitation, and mitral regurgitation were 4.6%/1.0%, 16.6%/4.1%, 13.9%/2.4%, 44.9%/12.8%, and 34.2%/11.3% at 3 years; 8.0%/3.3%, 30.6%/8.9%, 25.9%/6.0%, 71.2%/27.5%, and 60.7%/24.8% at 6 years; and 17.4%/7.6%, 35.5%/18.1%, 46.4%/10.7%, 88.5%/47.1%, and 87.6%/46.0% at 9 years, respectively. The false-positive group had a significantly higher risk for the development of moderate or severe aortic stenosis (HR 1.86, 95% CI 1.19–2.90), aortic regurgitation (HR 2.14, 95% CI 1.68–2.74), pulmonary regurgitation (HR 4.08, 95% CI 3.06–5.45), tricuspid regurgitation (HR 3.21, 95% CI 2.75–3.75), and mitral regurgitation (HR 2.69, 95% CI 2.29–3.16) than the true-negative group. Of note, this trend was also presented in the external validation set, and HRs of 1.76 to 3.15 were presented for each valvular heart disease.

## DISCUSSION

This was the first study to simultaneously develop DLMs for detecting multiple valvular diseases, and we found that these DLMs shared similar components to establish predictions. Therefore, they should be considered for integration into a single report for AI-ECG analysis. The sum of the positive prediction value and proportion of at least 1 significant echocardiographic finding was more than 50% in each DLM, which revealed the importance of an echocardiographic examination in those with any positive predictions by AI-ECG. Moreover, the significantly high risk of new-onset valvular diseases also reminds physicians to intensively monitor progression. These results emphasize the importance of AI-ECG as an initial screening test for managing valvular diseases.

This study achieved state-of-the-art performance in detecting aortic stenosis, [8, 9] aortic regurgitation, [10] and mitral regurgitation [11] compared to recently published retrospective studies using the same conditions. Moreover, we also demonstrated the feasibility of using AI-ECG for detecting pulmonary regurgitation (AUC >0.77) and tricuspid regurgitation (AUC >0.83), and all DLMs were not worse than the screening tests already implemented on a large scale, such as breast cancer screening (AUC = 0.78) [12] and fecal occult blood tests (AUC = 0.71) [13]. Since the characteristics of ECG are inexpensive, ubiquitous, and commonly used, asymptomatic valvular diseases may be detected early by AI-ECG with acceptable accuracy. In current clinical practice, patients are usually under the management of valvular heart disease when they become symptomatic. However, the symptoms are subjective, and a lack of symptoms is not benign. For example, sudden death without preceding symptoms occurred in 4.1% of patients with aortic stenosis [14]. Since the long-term results of prompt intervention in the asymptomatic stage are excellent, [15] AI-ECG may become popular for application in the early diagnosis of potential valvular diseases.

Our results emphasized that false-positives of valvular diseases by AI-ECG may be related to other echocardiographic abnormalities, and the correlation between predictions of each DLM was highly correlated. This correlation may not be sourced from the original relationship between each valvular disease, but it may be sourced by the similar ECG presentation in each valvular disease. The ECG findings may be nonspecific in valvular diseases. Due to the chronic pressure overload of the left ventricle, left ventricular hypertrophy secondary to aortic stenosis may be present on ECG [16]. Moreover, signs of left ventricular hypertrophy were also observed in aortic regurgitation and mitral regurgitation [17]. Right ventricular hypertrophy was also demonstrated on ECG in patients with tricuspid regurgitation and pulmonic regurgitation [18]. Moreover, because chronic valvular diseases are usually accompanied by cardiomyopathy, low ejection fraction or heart conduction abnormalities, [19] AI-ECG might use this information to construct valvular disease predictions. AI-ECG has already been validated to accurately detect echocardiographic abnormalities, such as left atrium enlargement, [6] low ejection fraction, [20] high left ventricle diameter, [21] and pulmonary hypertension [22]. Since the appropriate features may be learned by DLM on the basis of data rather than manual engineering, our AI-ECG maximally discovered the indirect relationship between ECG and valvular diseases. We can utilize this indirect relationship to identify patients with worse cardiac function and abnormal heart structure, and an echocardiography examination for positive cases may be cost-effective.

Previous studies have shown that AI-ECG has the ability to identify disease predictors [4]. This phenomenon was also observed in DLMs for detecting aortic stenosis [8] and mitral regurgitation, [11] and this study validated those findings and expanded the use of DLMs to more valvular diseases. We also further recognized that the positive predictions of our AI-ECG were based on a series of ECG changes, and patients with abnormal ECGs tended to have a higher risk of future cardiovascular events. A previous study mentioned that false-positive predictions of dyskalemia were formed by the combination of abnormal ECG rhythms, which led to a higher risk of mortality and hospitalization [23]. Therefore, even without considering the underlying heart structural changes implied by the abnormal ECG, these ECG rhythms might also need further intervention. For example, atrial fibrillation was associated with an increased risk of stroke and should be treated [24]. Moreover, left atrium enlargement has been found to be an independent risk factor for new-onset mitral regurgitation [25], and left atrium enlargement-related rhythms were also recognized to be related to stroke and prehypertension. Considering that screen-detected atrial fibrillation might have the same results as incidentally detected atrial fibrillation in regards to reducing the risk of stroke and death [26], physicians should attach importance to positive AI-ECG predictions to provide active management and not limit the use of Al-ECG to only identifying new-onset valvular diseases.

One of the most potentially impactful applications of AI-ECG in valvular heart diseases is opportunistic screening, which primarily originates from radiology. This refers to instances where patients occasionally benefit from radiologic imaging tests conducted for other reasons, thereby discovering potential signs of illness [27]. Previous cost-effectiveness study has found the advantages of using AI-ECG for opportunistic screening of asymptomatic left ventricular dysfunction [28], and it is plausible that valvular heart diseases could also be suitable for opportunistic screening. Considering the daily performance of up to three million ECG examinations worldwide [29], reanalyzing these already conducted tests with an AI model could potentially reduce the cost of screening for valvular heart diseases. Therefore, hospitals or clinics that routinely perform many ECG examinations should consider implementing AI-ECG to identify patients with valvular heart diseases, which may also contribute to delivering a higher standard of patient care.

Several limitations in this study should be mentioned. First, moderate and severe mitral stenosis was also present in more than 0.1% of patients. However, our echocardiographic database did not record it using structure format. Second, echocardiography was only conducted in patients with evidence of cardiovascular diseases in current clinical practice, and the prevalence of echocardiographic abnormalities in this study might be overestimated compared to the prevalence in asymptomatic people. However, this may not matter because positive ECGs still presented a higher risk of cardiovascular diseases and progression than negative ECGs. Third, some important clinical information, such as cardiac murmur, is lacking in this large-scale study due to the difficulty of reviewing all medical records. The performance of AI-ECG in asymptomatic people was unclear. Finally, there was still no clinical impact analysis of how many people may benefit from this screening system. An additional randomized controlled trial of our AI-ECG is currently planned.

## CONCLUSION

The AI-enabled 12-lead ECG may become a powerful screening tool for the detection of patients with moderate to severe valvular diseases. According to the existing evidence, an additional echocardiography examination for patients with a positive prediction by AI-ECG may be important. An appropriate treatment should be initiated once the true positive finding is validated, and unexpected significant echocardiographic findings may also remind physicians to manage the potential risk of cardiovascular diseases. The higher risk of new-onset valvular diseases should also be emphasized in patients with positive AI-ECG results to manage the related adverse events.

## METHODS

### Data source and population

This research was a retrospective study with ethics approval by the institutional review board without individual consent in the Tri-Service General Hospital, Taipei, Taiwan (IRB No. C202105049). Two separate institutions in the Tri-Service General Hospital system provided research data from Jan 2010 to Sep 2021. An academic medical center (Nei-Hu General Hospital) was named hospital A, and a community general hospital (Ting-Zhou Branch Hospital) was named hospital B in this study. Patients who had at least one ECG and echocardiography examination within 7 days were included.

**Figure 5:**
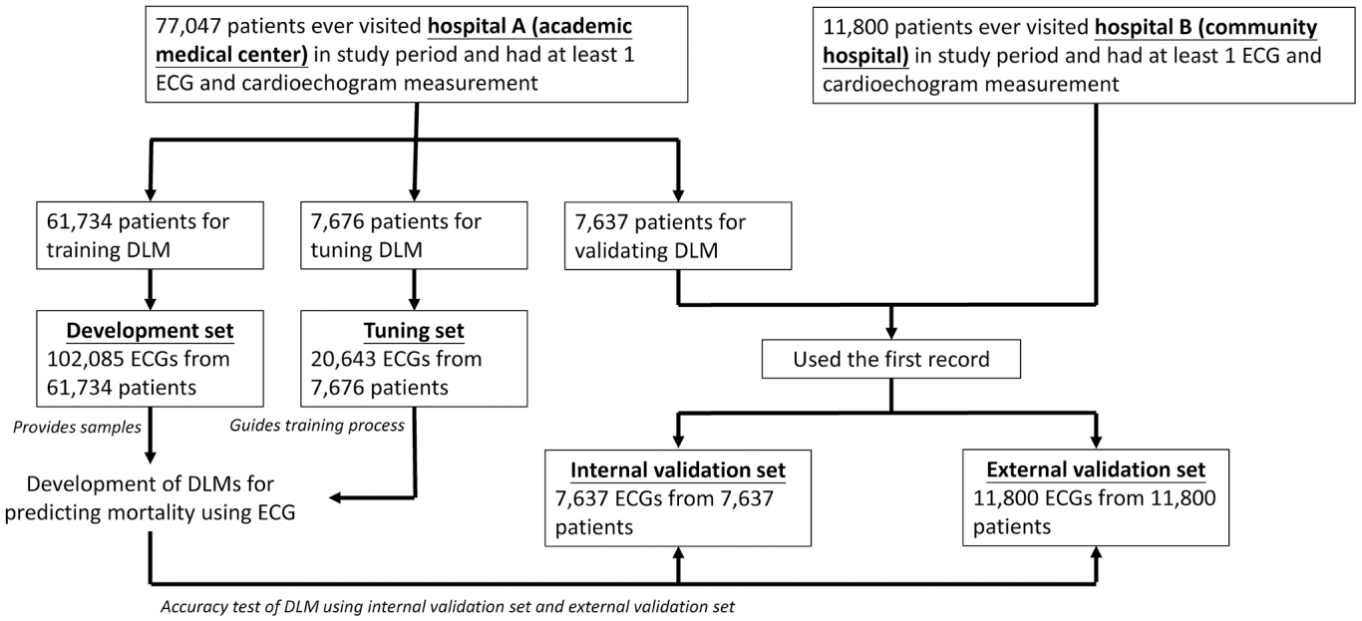
**Development, tuning, internal validation, and external validation set generation and ECG labeling of VHD.** Schematic of the dataset creation and analysis strategy, which was devised to assure a robust and reliable dataset for training, validating, and testing of the network. Once a patient’s data were placed in one of the datasets, that individual’s data were used only in that set, avoiding ‘cross-contamination’ among the training, validation, and test datasets. The details of the flow chart and how each of the datasets was used are described in the Methods.

Figure 5 shows the generation process of the development, tuning, and validation sets. There were 77,047 patients with ECGs and corresponding transthoracic echocardiography (TTE) annotations in this study period from hospital A. The 77,047 patients were divided into three groups: 102,085 ECG records from 61,734 patients as the development set, 20,643 ECG records from 7,676 patients as the tuning set, and 7,637 ECG records from 7,637 patients as the internal validation set. Importantly, we only used the first ECG in the validation sets to avoid patient dependency. The external validation set included 11,800 ECGs from 11,800 patients from hospital B. These validation sets were used to validate the performance of the DLM for predicting valvular diseases.

### Electrocardiographic signal

All 12-lead ECGs were recorded at the time of the acquisition in a Philips system^®^. There were 5,000 voltage–time trace signals for each lead (500 Hz sampling frequency for 10 seconds) to establish a 12 by 5,000 matrix as the DLM input. The Philips system^®^ also provided an automatic analysis for each ECG, and the statements were extracted by the basis of the key phrases as follows: sinus rhythm, atrial fibrillation/flutter, atrioventricular block, left bundle branch block, right bundle branch block, left atrial enlargement, left ventricular hypertrophy, prolonged QT interval, atrial premature complex, and ventricular premature complex.

### Present and new-onset valvular heart diseases

Comprehensive 2D echocardiograms were recorded at the date of the acquisition in a Philips image system®. TTE data were used to grade patients with minimal, mild, moderate, and severe valvular diseases using published guidelines [30]. The definition of moderate aortic stenosis was a jet velocity of 3.0–4.0 m/s, a mean gradient of 20–49 mmHg, or an *aortic* valve area (AVA) of 1.1–1.5 cm^2,^ and severe aortic stenosis was defined as a jet velocity ≥4.0 m/s, a mean gradient ≥40 mmHg, a DVI ≤0.25, or an AVA ≤1.0 cm^2^. The definition of moderate aortic regurgitation was a jet width of 25–64% of the left ventricular outflow tract (LVOT), a vena contracta of 0.3–0.6 cm, a regurgitant volume (RVol) of 30–59 mL/beat, a regurgitant fraction (RF) of 30–49% or an effective regurgitant orifice (ERO) of 0.10–0.29 cm^2,^ and severe aortic regurgitation was defined as a jet width ≥65% of the LVOT, a vena contracta >0.6 cm, a RVol ≥60 mL/beat, a RF ≥50%, or an ERO ≥0.3 cm^2^. The definition of moderate pulmonary regurgitation was a regurgitant fraction of 20–39%, and the definition of severe pulmonary regurgitation was a ratio of PR jet width >0.7, a pressure half-time of PR jet <100 msec, or a regurgitant fraction >40%. The definition of moderate tricuspid regurgitation was a vena contracta width of 0.3–0.69 cm, a proximal isovelocity surface area (PISA) radius of 6–9 mm, an ERO of 0.2–0.39 cm^2^, a regurgitant volume of 30–44 mL, or hepatic vein systolic flow blunting, and the definition of severe tricuspid regurgitation was a central jet ≥50% right atrial, a vena contracta width ≥0.7 cm, a PISA radius >9 mm, an ERO ≥0.40 cm^2^, a regurgitant volume ≥45 mL, or hepatic vein systolic flow reversal. The definition of moderate mitral regurgitation was a central jet MR of 20–39%, a vena contracta of 0.3–0.69 cm, a regurgitant volume of 30–59 mL, a regurgitant fraction of 30–49%, or an ERO 0.2–0.39 cm^2,^ and the definition of severe mitral regurgitation was a central jet MR >40%, a vena contracta ≥0.7 cm, a regurgitant volume ≥60 mL, a regurgitant fraction ≥50%, or an ERO ≥0.40 cm^2^ [30].

We followed patients with more than or equal to 2 TTE examinations for AI-ECG previvor analysis. For each analysis only patients with an initially minimal-to-mild corresponding valvular disease were used. This did not ensure that the included patients were completely normal; they may still have presented certain echocardiographic abnormalities, including other valvular diseases. The follow-up periods were started from the index TTE date to the corresponding events or the end of this study. Moreover, the follow-up data were censored at the last known TTE examination to limit bias from incomplete records.

### Study covariates

Patient characteristics, including demographics and medical comorbidities, were obtained from the electronic medical records. We used the International Classification of Diseases, Ninth Revision and Tenth Revision to define diabetes mellitus (DM), hypertension (HTN), hyperlipidemia (HLP), CKD, coronary artery disease (CAD), heart failure (HF), Afib, and chronic obstruction pulmonary disease (COPD), and the look-up tables were reported previously [31]. We also extracted echocardiography data, including ejection fraction (EF), left ventricle (end-diastole) (LV-D), left ventricle (end-systole) (LV-S), interventricular septum (IVS), left ventricular posterior wall (LVPW), left atrium (LA), aortic root (AO), right ventricle (RV), pulmonary artery systolic pressure (PASP), and pericardial effusion (PE), from TTE reports corresponding to the index ECG. The significant echocardiographic findings were defined by these parameters with appropriate clinical value.

### Deep learning and integration model

The architectures were consistent in DLMs for detecting each valvular disease developed previously [32]. In summary, it is a convolutional neural network using the 12 by 5,000 matrix of raw ECG signals as the input, and the output was a sigmoid output ranging from 0 to 1 to describe the binary outcomes. The training details were also mentioned previously. A batch size of 32, a weight decay of 10^−4^, and an initial learning rate of 0.001 with the Adam optimizer and standard hyperparameters were used, which decayed by a factor of ten each time the loss on the tuning set plateaued after an epoch. Early stopping was performed by saving the network after every epoch and selecting the saved network with the lowest loss on the tuning set. In this study, we trained 5 DLMs for detecting moderate-to-severe aortic stenosis, aortic regurgitation, pulmonary regurgitation, tricuspid regurgitation, and mitral regurgitation. To add age and sex to enhance the DLM performance, we used the XGB model to integrate DLM prediction and the training process mentioned previously [33]. We only performed prediction once using the best model based on the tuning set in the internal and external validation sets.

### Statistical analysis

Patient characteristics were expressed as numbers of patients, percentages, means, and standard deviations where appropriate. DLM performance for detecting each valvular disease was tested by receiver operating characteristic (ROC) curve analysis, and the outcome was defined as the moderate-to-severe group compared to the minimal-to-mild group. Indicators including the area under the ROC curve (AUC), sensitivity, specificity, positive predictive value (PPV), and negative predictive value (NPV) with 95% confidence interval (95% CI) were used to express the DLM accuracy. We also added age and sex as additional input features of the DLM to compare with previous studies.

We explored differences in ECG features of AI-identified positive and negative ECGs. The differences in echocardiographic characteristics were also compared in those two groups. We performed Kaplan–Meier survival analysis with the follow-up data for each valvular disease stratified by the DLM prediction. The data were censored on the basis of the most recent echocardiography. Cox proportional hazards models were also fit, and hazard ratios (HRs) with 95% CIs were used to compare the prognostic performances. All statistical analyses were carried out using the R language (version 3.4.4).

## References

[1] Nkomo VT, Gardin JM, Skelton TN, Gottdiener JS, Scott CG, Enriquez-Sarano M. Burden of valvular heart diseases: a population-based study. Lancet. 2006; 368:1005–11. 10.1016/S0140-6736(06)69208-8 16980116

[2] McDonagh TA, Metra M, Adamo M, Gardner RS, Baumbach A, Böhm M, Burri H, Butler J, Čelutkienė J, Chioncel O, Cleland JGF, Coats AJS, Crespo-Leiro MG, et al, and ESC Scientific Document Group. 2021 ESC Guidelines for the diagnosis and treatment of acute and chronic heart failure. Eur Heart J. 2021; 42:3599–726. 10.1093/eurheartj/ehab368 34447992

[3] Vahanian A, Beyersdorf F, Praz F, Milojevic M, Baldus S, Bauersachs J, Capodanno D, Conradi L, De Bonis M, De Paulis R, Delgado V, Freemantle N, Gilard M, et al, and ESC/EACTS Scientific Document Group. 2021 ESC/EACTS Guidelines for the management of valvular heart disease. Eur Heart J. 2022; 43:561–632. 10.1093/eurheartj/ehab395 34453165

[4] Attia ZI, Harmon DM, Behr ER, Friedman PA. Application of artificial intelligence to the electrocardiogram. Eur Heart J. 2021; 42:4717–30. 10.1093/eurheartj/ehab649 34534279 PMC8500024

[5] Kwon JM, Jeon KH, Kim HM, Kim MJ, Lim SM, Kim KH, Song PS, Park J, Choi RK, Oh BH. Comparing the performance of artificial intelligence and conventional diagnosis criteria for detecting left ventricular hypertrophy using electrocardiography. Europace. 2020; 22:412–9. 10.1093/europace/euz324 31800031

[6] Lou YS, Lin CS, Fang WH, Lee CC, Ho CL, Wang CH, Lin C. Artificial Intelligence-Enabled Electrocardiogram Estimates Left Atrium Enlargement as a Predictor of Future Cardiovascular Disease. J Pers Med. 2022; 12:315. 10.3390/jpm12020315 35207802 PMC8879964

[7] Hannun AY, Rajpurkar P, Haghpanahi M, Tison GH, Bourn C, Turakhia MP, Ng AY. Cardiologist-level arrhythmia detection and classification in ambulatory electrocardiograms using a deep neural network. Nat Med. 2019; 25:65–9. 10.1038/s41591-018-0268-3 30617320 PMC6784839

[8] Cohen-Shelly M, Attia ZI, Friedman PA, Ito S, Essayagh BA, Ko WY, Murphree DH, Michelena HI, Enriquez-Sarano M, Carter RE, Johnson PW, Noseworthy PA, Lopez-Jimenez F, Oh JK. Electrocardiogram screening for aortic valve stenosis using artificial intelligence. Eur Heart J. 2021; 42:2885–96. 10.1093/eurheartj/ehab153 33748852

[9] Kwon JM, Lee SY, Jeon KH, Lee Y, Kim KH, Park J, Oh BH, Lee MM. Deep Learning-Based Algorithm for Detecting Aortic Stenosis Using Electrocardiography. J Am Heart Assoc. 2020; 9:e014717. 10.1161/JAHA.119.014717 32200712 PMC7428650

[10] Sawano S, Kodera S, Katsushika S, Nakamoto M, Ninomiya K, Shinohara H, Higashikuni Y, Nakanishi K, Nakao T, Seki T, Takeda N, Fujiu K, Daimon M, et al. Deep learning model to detect significant aortic regurgitation using electrocardiography. J Cardiol. 2022; 79:334–41. 10.1016/j.jjcc.2021.08.029 34544652

[11] Kwon JM, Kim KH, Akkus Z, Jeon KH, Park J, Oh BH. Artificial intelligence for detecting mitral regurgitation using electrocardiography. J Electrocardiol. 2020; 59:151–7. 10.1016/j.jelectrocard.2020.02.008 32146201

[12] Pisano ED, Gatsonis C, Hendrick E, Yaffe M, Baum JK, Acharyya S, Conant EF, Fajardo LL, Bassett L, D'Orsi C, Jong R, Rebner M, and Digital Mammographic Imaging Screening Trial (DMIST) Investigators Group. Diagnostic performance of digital versus film mammography for breast-cancer screening. N Engl J Med. 2005; 353:1773–83. 10.1056/NEJMoa052911 16169887

[13] Haug U, Kuntz KM, Knudsen AB, Hundt S, Brenner H. Sensitivity of immunochemical faecal occult blood testing for detecting left- vs right-sided colorectal neoplasia. Br J Cancer. 2011; 104:1779–85. 10.1038/bjc.2011.160 21559011 PMC3111170

[14] Pellikka PA, Sarano ME, Nishimura RA, Malouf JF, Bailey KR, Scott CG, Barnes ME, Tajik AJ. Outcome of 622 adults with asymptomatic, hemodynamically significant aortic stenosis during prolonged follow-up. Circulation. 2005; 111:3290–5. 10.1161/CIRCULATIONAHA.104.495903 15956131

[15] Iung B, Gohlke-Bärwolf C, Tornos P, Tribouilloy C, Hall R, Butchart E, Vahanian A, and Working Group on Valvular Heart Disease. Recommendations on the management of the asymptomatic patient with valvular heart disease. Eur Heart J. 2002; 23:1253–66. 10.1053/euhj.2002.3320 12698958

[16] Kupari M, Turto H, Lommi J. Left ventricular hypertrophy in aortic valve stenosis: preventive or promotive of systolic dysfunction and heart failure? Eur Heart J. 2005; 26:1790–6. 10.1093/eurheartj/ehi290 15860517

[17] Gaasch WH, Meyer TE. Left ventricular response to mitral regurgitation: implications for management. Circulation. 2008; 118:2298–303. 10.1161/CIRCULATIONAHA.107.755942 19029478

[18] Glancy DL, Jain N, Jaligam VR, Ilie CC, Atluri P. Electrocardiogram in a woman with cor pulmonale. Proc (Bayl Univ Med Cent). 2011; 24:255–6. 10.1080/08998280.2011.11928728 21738303 PMC3124915

[19] Maganti K, Rigolin VH, Sarano ME, Bonow RO. Valvular heart disease: diagnosis and management. Mayo Clin Proc. 2010; 85:483–500. 10.4065/mcp.2009.0706 20435842 PMC2861980

[20] Chen HY, Lin CS, Fang WH, Lou YS, Cheng CC, Lee CC, Lin C. Artificial Intelligence-Enabled Electrocardiography Predicts Left Ventricular Dysfunction and Future Cardiovascular Outcomes: A Retrospective Analysis. J Pers Med. 2022; 12:455. 10.3390/jpm12030455 35330455 PMC8950054

[21] Chen HY, Lin CS, Fang WH, Lee CC, Ho CL, Wang CH, Lin C. Artificial Intelligence-Enabled Electrocardiogram Predicted Left Ventricle Diameter as an Independent Risk Factor of Long-Term Cardiovascular Outcome in Patients With Normal Ejection Fraction. Front Med (Lausanne). 2022; 9:870523. 10.3389/fmed.2022.870523 35479951 PMC9035739

[22] Liu CM, Shih ESC, Chen JY, Huang CH, Wu IC, Chen PF, Higa S, Yagi N, Hu YF, Hwang MJ, Chen SA. Artificial Intelligence-Enabled Electrocardiogram Improves the Diagnosis and Prediction of Mortality in Patients With Pulmonary Hypertension. JACC Asia. 2022; 2:258–70. 10.1016/j.jacasi.2022.02.008 36338407 PMC9627911

[23] Lin C, Chau T, Lin CS, Shang HS, Fang WH, Lee DJ, Lee CC, Tsai SH, Wang CH, Lin SH. Point-of-care artificial intelligence-enabled ECG for dyskalemia: a retrospective cohort analysis for accuracy and outcome prediction. NPJ Digit Med. 2022; 5:8. 10.1038/s41746-021-00550-0 35046489 PMC8770475

[24] Gladstone DJ, Spring M, Dorian P, Panzov V, Thorpe KE, Hall J, Vaid H, O'Donnell M, Laupacis A, Côté R, Sharma M, Blakely JA, Shuaib A, et al, and EMBRACE Investigators and Coordinators. Atrial fibrillation in patients with cryptogenic stroke. N Engl J Med. 2014; 370:2467–77. 10.1056/NEJMoa1311376 24963566

[25] Park SM, Park SW, Casaclang-Verzosa G, Ommen SR, Pellikka PA, Miller FA Jr, Sarano ME, Kubo SH, Oh JK. Diastolic dysfunction and left atrial enlargement as contributing factors to functional mitral regurgitation in dilated cardiomyopathy: data from the Acorn trial. Am Heart J. 2009; 157:762.e3–10. 10.1016/j.ahj.2008.12.018 19332207

[26] Jones NR, Taylor CJ, Hobbs FDR, Bowman L, Casadei B. Screening for atrial fibrillation: a call for evidence. Eur Heart J. 2020; 41:1075–85. 10.1093/eurheartj/ehz834 31811716 PMC7060457

[27] Berland LL, Silverman SG, Gore RM, Mayo-Smith WW, Megibow AJ, Yee J, Brink JA, Baker ME, Federle MP, Foley WD, Francis IR, Herts BR, Israel GM, et al. Managing incidental findings on abdominal CT: white paper of the ACR incidental findings committee. J Am Coll Radiol. 2010; 7:754–73. 10.1016/j.jacr.2010.06.013 20889105

[28] Liu WT, Hsieh PH, Lin CS, Fang WH, Wang CH, Tsai CS, Hung YJ, Hsieh CB, Lin C, Tsai DJ. Opportunistic Screening for Asymptomatic Left Ventricular Dysfunction With the Use of Electrocardiographic Artificial Intelligence: A Cost-Effectiveness Approach. Can J Cardiol. 2023. [Epub ahead of print]. 10.1016/j.cjca.2023.11.044 38092190

[29] Shenasa M. Learning and teaching electrocardiography in the 21^st^ century: A neglected art. J Electrocardiol. 2018. [Epub ahead of print]. 10.1016/j.jelectrocard.2018.02.007 29499830

[30] Baumgartner H, Hung J, Bermejo J, Chambers JB, Edvardsen T, Goldstein S, Lancellotti P, LeFevre M, Miller F Jr, Otto CM. Recommendations on the Echocardiographic Assessment of Aortic Valve Stenosis: A Focused Update from the European Association of Cardiovascular Imaging and the American Society of Echocardiography. J Am Soc Echocardiogr. 2017; 30:372–92. 10.1016/j.echo.2017.02.009 28385280

[31] Liu WC, Lin CS, Tsai CS, Tsao TP, Cheng CC, Liou JT, Lin WS, Cheng SM, Lou YS, Lee CC, Lin C. A deep learning algorithm for detecting acute myocardial infarction. EuroIntervention. 2021; 17:765–73. 10.4244/EIJ-D-20-01155 33840640 PMC9724911

[32] Lee CC, Lin CS, Tsai CS, Tsao TP, Cheng CC, Liou JT, Lin WS, Lee CC, Chen JT, Lin C. A deep learning-based system capable of detecting pneumothorax via electrocardiogram. Eur J Trauma Emerg Surg. 2022; 48:3317–26. 10.1007/s00068-022-01904-3 35166869

[33] Liu WT, Lin CS, Tsao TP, Lee CC, Cheng CC, Chen JT, Tsai CS, Lin WS, Lin C. A Deep-Learning Algorithm-Enhanced System Integrating Electrocardiograms and Chest X-rays for Diagnosing Aortic Dissection. Can J Cardiol. 2022; 38:160–8. 10.1016/j.cjca.2021.09.028 34619339

